# Frequency of *FLT3* Internal Tandem Duplications in Adult Syrian Patients with Acute Myeloid Leukemia and Normal Karyotype

**DOI:** 10.31557/APJCP.2021.22.10.3245

**Published:** 2021-10

**Authors:** Ismael F Al-Arbeed, Abdulsamad Wafa, Faten Moassass, Bassel AL-Halabi, Walid Al-Achkar, Imad Abou-Khamis

**Affiliations:** 1 *Department of Microbiology, Hematology and Immunology, Faculty of Pharmacy, Damascus University, Ministry of High Education, Damascus, Syria. *; 2 *Department of Molecular Biology and Biotechnology, Human Genetics Division, Atomic Energy Commission, Damascus, Syria. *

**Keywords:** Acute myeloid leukemia, normal karyotype, FLT3 internal tandem duplications, prognostic factors

## Abstract

**Objective:**

Activating mutations of the fms-like tyrosine kinase 3 gene (*FLT3*) by internal tandem duplications (ITDs) in the juxtamembrane domain (JMD) have been reported in ~30% of adult acute myeloid leukemia (AML) patients with cytogenetically normal karyotype (CN). However, *FLT3*/ITD mutations are frequently accompanied with leukocytosis, high percentage of blasts in bone marrow (BM), and increased the risk of treatment failure in AML patients. *FLT3*-ITD mutated AML patients mainly with normal karyotype have higher relapse probability and shorter duration of complete remission (CR) after chemotherapy, so *FLT3*-ITD mutation is considered as an independent poor prognostic factor in AML.

**Methods:**

*FLT3*-ITD and *FLT3*-KTD were studied by polymerase chain reaction (PCR) and restriction fragment length polymorphism- PCR (RFLP-PCR) in 44 adults AML patients with cytogenetically normal karyotype (AML-CN) at diagnosis to characterize *FLT3* status. The results were correlated with the prognostic factors.

**Results:**

In this study, *FLT3*-ITD mutations were identified in 7 (15.9%) of the 44 AML-CN patients. Among the 7 patients with *FLT3*/ITD mutations, 6 patients revealed a typical ITDs mutation (fragment size was 329 bp) and one patient showed untypical ITD mutation (fragment size was ~400 bp). Whereas 37 patients (61.7%) were *FLT3*-ITD. None of all AML-CN patients examined showed *FLT3*-KTD mutations.

**Conclusions:**

Our results support that *FLT3*-ITD are independent adverse prognostic factors for elderly AML-CN patients and are associated with low overall survival (OS), low rate of CR, high relapse rate (RR), and high percentage of BM blast at diagnosis. We concluded, *FLT3* mutation analysis should be performed as a routine test in AML-CN patients.

## Introduction

In patients with acute myeloid leukemia (AML) genetic diagnostics was performed in the past mainly by cytogenetics and molecular cytogenetics. In recent years also tumor markers were added, which can be uncovered only by molecular genetic methods (Kirtonia et al., 2020). 

The fms-like tyrosine kinase 3 (*FLT3*) gene encodes a class III receptor tyrosine kinase for the *FLT3* ligand, which is normally expressed in CD34+ hematopoietic stem/progenitor cells and plays a fundamental role in both normal and leukemic hematopoiesis (Stirewalt et al., 2003). Internal tandem duplications (ITDs) of the *FLT3* gene (*FLT3*-ITDs) represent one of the most common molecular abnormalities in patients with AML, detectable in around 25%-30% of all patients (Schnittger et al., 2002; Patnaik 2018). ITDs consist of in-frame insertions of duplicated sequences localized in the juxtamembrane domain (JMD) of the *FLT3* molecule. Their presence results in a constitutive, ligand independent activation of the tyrosine kinase activity of the *FLT3* receptor; this is responsible for abnormal proliferation and differentiation of leukemia stem cells (Stirewalt et al., 2003). Constitutive activation of kinase domain is due to disruption of auto-inhibitory interaction between JMD and the activation loop in AML, which normally stabilizes inactive kinase, and at the same time protects ATP binding pocket (Griffith et al., 2004; Chan 2011). Also, *FLT3*-ITDs protect leukemia cells from damaging chemotherapeutic agents (Lagunas-Rangel and Chávez-Valencia, 2017).

Presence of *FLT3*-ITDs has been associated with an increase an initial peripheral white blood cell (WBC) count, a high percentage of bone marrow blast cells, reduced disease-free survival (DFS) and overall survival (OS), and increased relapse rate (RR) with an overall adverse prognosis. However, rate of complete remission (CR) was not significantly affected (Kottaridis et al., 2001; Yanada et al. 2005; Canaani et al., 2018). Thus, a prognostic significance of *FLT3*/ITDs has been suggested (Stirewalt et al., 2006) and according to National Comprehensive Cancer Network and European LeukemiaNet (ELN) 2017, cases with *FLT3*-ITD mutation plus cytogenetically normal karyotype have a poor prognosis. 

Whereas, the less frequent *FLT3*-TKD mutations are of unclear prognostic relevance have been observed in ~7% of AML patients (Bacher et al., 2008; Yamamoto et al., 2001; Kim 2010). However, the incidence of ITD and TKD mutations in *FLT3* vary slightly according to age, clinical risk groups, and cytogenetic profile (Levis, 2013). Moreover, adult AML patients usually have a higher prevalence of *FLT3*-ITDs than pediatric patients. This observation may partially explain why adult AML has a poorer clinical outcome than pediatric AML (Gregory et al., 2009). Many clinical studies have shown that patients with an ITD at diagnosis have frequent disease relapses and a short duration of survival when compared to patients without an ITD (Schnittger et al., 2002; Kim et al., 2004).

In this study, we investigated both the prevalence and prognostic significance of *FLT3*-ITDs in adult Syrian AML patients with cytogenetically normal (AML-CN) and could show, it has an impact on the therapy outcomes of AML.

## Materials and Methods

*Subjects*

The present study comprised 44 newly patients diagnosed with de novo AML between October 2018 and February 2020 were included in this study. Patients without previous treatment were included in the study; patients with normal karyotype were selected for molecular analysis and patients with history of exposure to chemotherapy/radiotherapy, and secondary AML patients, were excluded. AML diagnosis was made according to French-American-British (FAB) classification. Their initial bone marrow (BM) or peripheral blood (PB) samples were collected for use in the study. Patients consisted of 23 men and 21 women; the median age was 35.3±12.4 years (range, 18-77 years) (Table 1). This study was approved by the Ethics Committee in Syrian Ministry of High Education and written informed consent was obtained from all the participants.

*Treatment protocol*

The majority of patients received (3+7) standard induction chemotherapy, which consisted of daunorubicin at 45 mg/m^2^ for 3 days and cytarabine at 100-200 mg/m2 for 7 days, followed by high doses of a cytarabine-based consolidation phase (cytarabine at mg/m2 3 every 12 h for 3 days, repeated for 2 to 3 cycles). Patients with acute promyelocytic leukemia (M3) received all-trans retinoic acid plus anthracycline. Patients received conventional induction chemotherapy and were followed for 14 months. BM aspiration was performed between 21 and 28 days after initiation of chemotherapy. The patients were followed up once every 3 months with clinical examination and complete blood counts. A BM aspiration was performed if there was any suggestion of relapse on clinical examination or peripheral smear. 

*Cytogenetic and molecular cytogenetic analyses*

Chromosome analysis using GTG-banding was performed on BM sample prior to chemotherapy acc. to standard protocols (AL-Achkar et al., 2007). Fluorescence in situ hybridization (FISH) using specific probes to detect translocations t(8;21), t(15;17), t(16;16), t(12;21), and deletion del(13q), were performed with standard method to excluded patients with chromosomal abnormalities, as previously reported (AL-Achkar et al., 2007). 

*Sample collection*

Genomic DNA was isolated from PB or BM samples from de novo AML patients using the QIAamp DNA Blood Mini kit (Qiagen, Germany) according to the manufactures instructions and was stored at -20°C. The total DNA of each sample was measured by using a spectrophotometer followed by quantity ultraviolet light absorbance

*Analysis of the FLT3-ITD mutation*

Exons 14 and 15 of the *FLT3*-ITD mutation were amplified using specific forward primer 5’-GCAATTTAGGTATGAAAGCCAGC-3’ and reverse primer 5’-CTTTCAGCATTTTGACGGCAACC-3’ (Rezaei et al., 2017). The PCR reaction was performed in a total volume of 50 μl containing 200 ng of genomic DNA, 10xPCR buffer (100 mM Tris-HCl, pH 8.8, 500 mM KCl), 2 mM MgCl2, 200 μM dNTPs, 10 pM of each primer, and 1 U of Taq DNA polymerase. PCR conditions included initial denaturation at 95°C for 5 min followed by 30 cycles of 94°C for 30 s, 56°C for 30 s, and 72°C for 45 s with a final extension at 72 °C for 5 min. PCR reaction was conducted in a PCR T100 thermocycler (Applied Biosystems, USA). The 329-bp PCR products were run on 3% agarose gel stained with DNA SafeStain Dye and visualized under UV light. Samples with additional longer PCR products were identified as *FLT3*-ITD+. All mutant samples were verified by direct sequencing using the ABI Prism 310 genetic analyzer (Applied Biosystems, Foster City, CA, USA). The cycle-sequencing reaction was performed in a 10-μl volume containing 1 μl of the terminator ready reaction, 5 pmol of either the forward or reverse primer and 10 ng of purified PCR product (ExoSAP-IT kit; Amersham BioSciences, Piscataway, NJ, USA). The thermal cycle protocol was 95˚C for 4 min followed by 30 cycles at 96˚C for 10 sec, 50˚C for 5 sec and 60˚C for 4 min (ABI GeneAmp PCR System 9700, Applied Biosystems). Centri-Sep columns (Princeton Separations, Adelphia, NJ, USA) were used for the effective and reliable removal of excess dye terminators (DyeEx 2.0, Qiagen, Germany) from completed DNA sequencing reactions. Data were compared and aligned with different sequences using the NCBI BLAST Assembled Genomes tool (http://blast.ncbi.nlm.nih.gov/Blast.cgi).

*Analysis of the FLT3-TKD mutation*

For detection of the *FLT3*-TKD mutation, the specific forward primer 5’-CCGCCAGGAACGTGCTTG-3’ and reverse primer 5’-GCAGCCTCACATTGCCCC-3’ were used (Rezaei et al., 2017). The PCR reaction was performed in a total volume of 15 μl with similar reagents as used for the *FLT3*-ITD mutation, except for the primers. PCR conditions were also the same, except for the annealing temperature, which was 65 °C for 30 s. The amplification reaction was conducted in a PCR T100 thermocycler (Applied Biosystems). The 119-bp PCR products were then digested with 2 U of EcoRV at 37°C for 17 h, run on 3% agarose gel stained with DNA SafeStain Dye, and visualized under UV light. The presence of an undigested PCR product was an indication of a mutant sample.

*Statistical analysis*

The comparison of qualitative data such as age, WBC count, platelet count, hemoglobin level and blast count percentage between *FLT3*-ITD+ and *FLT3*-ITD- patients were statistically evaluated using Fisher exact and chi-square tests. OS and DFS were estimated for patients who received at least one induction course of therapy using the Kaplan-Meier method. p<0.05 was considered to be of statistical significance. All analyses were performed using SPSS Statistics 19 software (SPSS, Chicago, IL, USA).

## Results

Table 2 summarizes the characteristics of the newly diagnosed AML-CN patients included in the study. Of the 44 AML patients studied, 23 were males (52.2%) and 21 were females (47.7%); 7 out 44 cases (3 males and 4 females) were positive for *FLT3*-ITD+ mutations (15.9%), 6 out 7 *FLT3*-ITD+ patients (fragment size was more than 329 bp) and one out 7 *FLT3*/ITD+ patients showed untypical ITDs mutation (fragment size was ~400 bp) (Alarbeed et al., 2021); whereas 37 patients (61.7%) were *FLT3*-ITD- (fragment size was 329 bp). None of all AML-CN patients examined showed *FLT3*-KTD mutations.

There were no significant differences between *FLT3*-ITD+ and *FLT3*-ITD-patients with respect to sex, WBC, hemoglobin, platelet counts, and AML FAB subtypes (Table 2). However, patients with *FLT3*-ITD+ was older than *FLT3*-ITD- patients (47.9±14.4 vs. 32.8±10.4; p=0.002) and had high percentage of bone marrow blasts compared with *FLT3*-ITD- patients (78.3±13.9 vs. 67.3±13.6; p=0.07).

**Figure 1:**
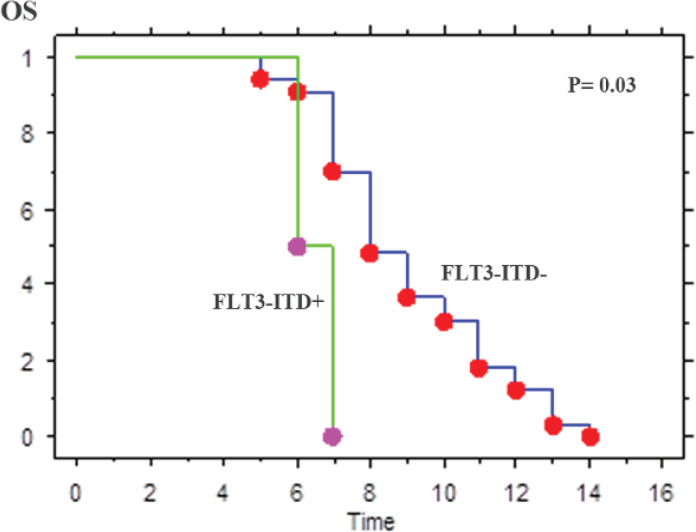
Kaplan-Meier AAnalysis of OS of Patients with AML- CN According to the FLT3-ITD Status

Of the 44 patients who received standard induction chemotherapy, 39 patients (88.6%) achieved CR. The CR rate was significantly affected in patients with *FLT3*-ITD+ compared *FLT3*-ITD- (28.6% vs. 75.6%, p˂0.01) (Table 3). However, of the patients who achieved CR, patients with *FLT3*-ITD+ had a higher RR and a low OS than patients with *FLT3*-ITD- (RR: 57.1% vs. 13.5%, p=˂0.01), (OS rate: 4 vs. 8.2 months, p = 0.03) (Table 4, Figure 1). However, these differences were statistically significant. *FLT3*-ITD+ was a sole independently poor prognostic factor for DFS and OS.

**Table 1:** Demographic and Laboratory Data of Syrian AML Cytogeneticlly Nnormal Patients

Parameters	Value
Gender	
Male	23 (52.2%)
Female	21 (47.7%)
Sex ratio (M/F)	1.1
Age (median, range)	35.3±12.4
FAB classification	
M1	7 (15.9%)
M2	8 (18.1%)
M3	5 (11.3%)
M4	13 (29.5%)
M5	10 (22.7%)
M6	1 (2.2%)
WBC, x 10^9^/l (median, range)	44 (0.8-300)
Hb, g/dl (median, range)	8.7 (3.5-16.7)
Plt, x 10^9^/l (median, range)	78.3 (17-309)
BM Blasts,%	69.6 (42-94)

FAB, French-American-British classifications; WBC, White blood cells; Hb, hemoglobin; Plt, Platelets; BM, bone marrow

**Table 2:** Clinical Patients Characteristics According to FLT-3 Status in Syrian AML Normal Cytogeneticlly Patients

Features	*FLT3*-ITD+	*FLT3*-ITD−	P value
Patients no (%)	7 (15.9%)	37 (48.09%)	
Gender			
Male	3 (42.9%)	20 (54%)	0.6
Female	4 (57.1%)	17 (46%)	
Sex ratio (M/F)	0.75	1.1	
Age (years)			
Mean	47.9±14.4	32.8±10.4	0.002
range	18-64	18-57	
WBC, x 10^9^/l			
Median	41.8±21.3	44.6±65.1	0.9
Range	4.5-64	0.8-300	
Hb (g/dl)			
Median	8.6±1.4	8.7±2.6	0.9
Range	6-10.5	3.5-16.7	
Plt x 10^9^/l			
Median	81.4±29.6	77.5±55.9	0.9
Range	32-112	17-309	
BM Blasts,%			
Median	78.3±13.9	67.3±13.6	0.07
Range	60-94	42-90	
FAB:M4&M5/others	4 (57.1%)	19 (51.3%)	0.8

FAB, French-American-British classifications; WBC, White blood cells; Hb, hemoglobin; Plt, Platelets; BM, bone marrow. P ˂ 0.05 is considered significant

**Table 3:** Outcome Data According to *FLT*/ITD Mutational Status in Syrian AML-CN Patients

Groups	Fate			P
	CR	RD	ID	
FLT3-ITD+	2/7 (28.6%)	4/7 (57.1%)	1/7 (14.3%)	˂0.01
FLT3-ITD-	28/37 (75.6%)	5/37 (13.5%)	4/37 (10.8%)	

CR, complete remission; RD, resistant disease; ID, induction death.

**Table 4:** Outcome Data According to FLT/ITD Mutational Status in Syrian AML-CN Patients

Mutational status	Mean OS (months)	P	Mean DFS (months)	P
FLT3-ITD-	8.2	0.03	8.8	0.02
FLT3-ITD+	4.0		3.3	

OS, overall survival; DFS, disease-free survival

**Table 5:** Prevalence of FLT3-ITD Mutation in AML in Various Studies

Study	Country	Number of AML cases	Total prevalence %
Present study	Syria	44	15.9
Thiede et al. (2002)	Germany	979	20.4
Elyamany et al. (2014)	Saudi Arabia	97	14.4
Aly et al. (2012)	Egypt	39	15.4
Pazhakh et al. (2011)	Iran	131	16
Ishfaq et al. (2012)	Pakistan	30	13.3
Gari et al. (2008)	Saudi Arabia	129	11.6
Sheikhha et al. (2003)	Iran	80	10
Xu et al. (2012)	China	216	20.8
Wang et al. (2005)	China	76	19.7
Al-Tonbary et al. (2009)	Egypt	30	20
Suzuki et al. (2007)	Japan	60	20
Wang et al. (2010)	China	143	25.9
Auewarakul et al. (2005)	Thiland	256	27.3
Fröhling et al. (2002)	USA	224	32

## Discussion

We evaluated the prevalence and prognostic *FLT3*-ITD+ in 44 Syrian adult patients with AML-CN newly diagnosed. 

The presence of *FLT3*-ITD+ had received significant attention due to contribute to disease progression and poor prognosis for patients. The clinical significance of *FLT3*-ITD+ has been clearly demonstrated in previous studies (Levis, 2013; Thiede et al., 2002). Adult studies have shown a prevalence of 25–30% for the *FLT3*-ITD+ in AML patients who have no cytogenetic abnormalities (Schnittger et al., 2002; Patnaik 2018; Griffith et al., 2004) and ~7% for *FLT3*-KTD point mutation of the activation loop domain (Bacher et al., 2008; Yamamoto et al., 2001; Kim 2010). The incidence of *FLT3*-ITD+ in the current study was 15.9%, which is obviously similar incidences having been previously reported in some studies (Table 5) (Elyamany et al., 2014; Aly et al., 2012; Pazhakh et al., 2011). Several studies observed a lower incidence of *FLT3*-ITD+ than in our study (Elyamany et al., 2014; Aly et al., 2012; Pazhakh et al., 2011; Ishfaq et al., 2002; Gari et al., 2008; Sheikhha et al., 2003). Various studies have detected a high occurrence of *FLT3*-ITD+ (Thiede et al., 2002; Xu et al., 2012; Wang et al., 2010; Al-tonbary et al., 2009; Suzuki et al., 2007; Wang et al., 2005; Auewarakul et al., 2005; Fröhling et al., 2002). However, those studies evaluated AML with or without abnormal karyotype. The differences in these results may additionally be explained due to sample sizes, geographic and ethnic background of the studied populations. 

The clinical impact of the characteristics of patients with and/or without *FLT3*-ITD demonstrated no statistically significant difference between *FLT3*-ITD+ and *FLT3*-ITD− patients regarding to a gender, our findings which are an agreement with those have been reported (Bao et al., 2006; Zwaan et al., 2003). Clinically, AML patients with *FLT3*-ITD+ tend to have higher WBC counts and an increased percentage of leukemic blasts (Kottaridis et al., 2001; Yanada et al., 2005; Canaani et al., 2018). In our study, a positive association has been found between *FLT3*-ITD+ mutation versus BM blasts count (Kottaridis et al., 2001; Yanada et al., 2005; Canaani et al., 2018). 

Regarding to CR rate, presence of the *FLT3*-ITD+ mutation did not appear to influence the achievement of CR (Kottaridis et al., 2001; Yanada et al., 2005; Canaani et al., 2018). In the other hand, many studies have demonstrated that *FLT3*-ITD+ patients had low CR rate, short OS, and high cumulative incidence of relapse after chemotherapy combined with *FLT3* inhibition (Chen et al., 2020; Meshinchi et al., 2001; Al-Mawali A et al., 2013). In our study, CR rate was statistically lower in *FLT3*-ITD+ (28.6%) than that in *FLT3*-ITD− (78.4%) subjects (P ˂0.01) our results in agreement with previously findings (Chen et al., 2020; Meshinchi et al., 2001; Al-Mawali A et al., 2013). 

Although the clinical significance of this *FLT3* mutation especially in AML-CN not clear yet. However, several studies showed that *FLT3*-ITD+ mutation is a strong adverse prognostic factor in AML patients (Stirewalt et al., 2008; Kiyoi and Naoe 2006; Zheng and Small 2005) with reduced DFS and OS and increased RR (Kottaridis et al., 2001; Yanada et al., 2005; Canaani et al., 2018). In our study, median OS was 4.0 months for *FLT3*-ITD+ patients and 8.2 months for *FLT3*-ITD- patients (*p*= 0.03), and DFS was also worse for *FLT3*-ITD+ than *FLT3*-ITD- patients (*p*= 0.02) because of a higher RR in *FLT3*-ITD+ patients. Also, high leucocytes count, high blast cells count in peripheral blood and resistance to therapy confers a poor prognosis. This has led to the development of a number of small molecule tyrosine kinase inhibitors (TKI) with activity against *FLT3* (Small 2006; Leung et al., 2013). Moreover, patients with low or absent levels of *FLT3*-ITD, consistent with homozygosity for the *FLT3*-ITD allele, appear to have a particularly dismal outcome (Thiede et al., 2002).

A failure to achieve post-induction remission was observed in 57.1% (4/7) of evaluable pediatric patients with *FLT3*-ITD+, as opposed to 13.5% (5/37) of *FLT3*-ITD- patients (P= ˂0.01), our findings in accordance with Kumiko et al. (Kumiko et al., 2005). Most of patients with *FLT3*-ITD+ were found to be resistant to initial chemotherapy and failed to achieve CR (Xu et al., 2000; Arrigoni et al., 2003). Older patients (age less than 60 years) with *FLT3*-ITD+ mutation have a significantly association with increased RR (Kottaridis et al., 2003).

Schnittger et al., (2018) could show that decreased *FLT3*-ITDs positively correlates with older age. However, other studies that did not reveal any age-dependency of *FLT3*-ITD, being performed in cohorts including other cytogenetic groups, smaller patient numbers of AML-CN, or restriction to patients up to 60 years of age (Thiede et al., 2002; Fröhling et al., 2002; Gale et al., 2005). In our study, patients who had *FLT3*-ITD+ were older than *FLT3*-ITD- (P=0.002).

In Conclusion, we report here for the first time the frequency and prognosis of the presence of *FLT3*-ITD+ mutations in adult Syrian patients with AML-CN. The frequency of *FLT3* mutations in our study was lower (15.9%) than in previous studies; however, some reports agree with our observation, and that these mutations are an important adverse prognostic factor. Overall, this report supports the view that *FLT3*-ITD+ is a strong prognostic factor in AML patients and is associated with low CR, high RR, resistance to therapy, low OS, low DFS and confers a poor prognosis. Thus, *FLT3* mutation analysis should be performed as a routine test in AML-CN patients. 

## Author Contribution Statement

IA provided all cases, a clinical data and a chemotherapy plan; AW and WA did primary cytogenetic and main part of the FISH-tests; FA and BA performed the molecular cytogenetic analyses; IA scientific supervisor of the IA student and put of the work plan. IA and AW drafted the paper and all authors worked on the final version of the paper. All authors read and approved the final manuscript.

## References

[B1] Alarbeed IF, Wafa A, Moassass F (2021). De novo adult acute myeloid leukemia with two new mutations in juxtatransmembrane domain of the FLT3 gene- A case report. J Med Case Rep.

[B2] AL-Achkar W, Wafa A, Nweder MS (2007). A complex translocation t(5;9;22) in Philadelphia cells involving the short arm of chromosome 5 in a case of chronic myelogenous leukemia. J Exp Clin Cancer Res.

[B3] Al-Mawali A, Gillis D, Lewis I (2013). Characteristics and prognosis of adult acute myeloid leukemia with internal tandem duplication in the FLT3 gene. Oman Med J.

[B4] Al-tonbary Y, Mansour AK, Ghazy H (2009). Prognostic significance of foetallike tyrosine kinase 3 mutation in Egyptian children with acute Leukaemia. Inter J Lab Hemat.

[B5] Aly R, Shahin D, Azmy E (2012). Prognostic significance of FLT3 internal tandem duplication in Egyptian acute myeloid leukemia and normal cytogenetics. Comp Clin Pathol.

[B6] Arrigoni P, Beretta C, Silvestri D (2003). FLT3 internal tandem duplication in childhood acute myeloid leukaemia: association with hyperleucocytosis in acute promyelocytic leukaemia. Br J Haematol.

[B7] Auewarakul CU, Sritana N, Limwongse C (2005). Mutations of the FLT3 gene in adult acute myeloid leukemia: determination of incidence and identification of a novel mutation in a Thai population. Cancer Genet Cytogenet.

[B8] Bacher U, Haferlach C, Kern W (2008). Prognostic relevance of FLT3-TKD mutations in AML: the combination matters – an analysis of 3082 patients. Blood.

[B9] Bao L, Wang X, Ryder J (2006). Prospective study of 174 de novo acute myelogenous leukemias according to the WHO classification: subtypes, cytogenetic features and FLT3 mutation. Eur J Haematol.

[B10] Canaani J, Labopin M, Huang XJ (2018). T-cell replete haploidentical stem cell transplantation attenuates the prognostic impact of FLT3-ITD in acute myeloid leukemia: a report from the Acute Leukemia Working Party of the European Society for Blood and Marrow Transplantation. Am J Hematol.

[B11] Chen F, Sun J, Yin C (2020). Impact of FLT3-ITD allele ratio and ITD length on therapeutic outcome in cytogenetically normal AML patients without NPM1 mutation. Bone Marrow Transplant.

[B12] Chan PM (2011). Differential signaling of Flt3 activating mutations in acute myeloid leukemia: a working model. Protein Cell.

[B13] Elyamany G, Awad M, Fadalla K (2014). Frequency and Prognostic Relevance of FLT3 Mutations in Saudi Acute Myeloid Leukemia Patients. Adv Hematol.

[B14] Kim HJ (2010). Mutations in AML with a normal karyotype: NPM1 and FLT3-ITD, ready to use as a key prognosticator?. Korean J Hematol.

[B15] Kim YK, Lee JJ, Lee YR (2004). The presence of FLT3/ ITD mutations is an independent prognostic factor in acute myeloid leukemia patients with normal karyotype. Blood.

[B16] Kiyoi H, Naoe T (2006). Biology, clinical relevance, and molecularly targeted therapy in acute leukemia with FLT3 mutation. Int J Hematol.

[B17] Kirtonia A, Pandya G, Sethi G (2020). A comprehensive review of genetic alterations and molecular targeted therapies for the implementation of personalized medicine in acute myeloid leukemia. J Mol Med (Berl).

[B18] Kumiko O, Sakurako Y, Sumio K (2005). Activating mutations of FLT3 gene in childhood acute myeloid leukemia and their applications for detection of minimal residual disease. Japan J Pediatr Hematol.

[B19] Kottaridis PD, Gale RE, Frew ME (2001). The presence of a FLT3 internal tandem duplication in patients with acute myeloid leukemia (AML) adds important prognostic information to cytogenetic risk group and response to the first cycle of chemotherapy: analysis of 854 patients from the United Kingdom Medical Research Council AML 10 and 12 trials. Blood.

[B20] Kottaridis P, Gale RE, Linch DC (2003). FLT3 mutations and leukemia. Br J Haematol.

[B21] Fröhling S, Schlenk RF, Breitruck J (2002). Prognostic significance of activating FLT3 mutations in younger adults (16 to 60 years) with acute myeloid leukemia and normal cytogenetics: a study of the AML Study Group Ulm. Blood.

[B22] Gale RE, Hills R, Pizzey AR (2005). Relationship between FLT3 mutation status, biologic characteristics, and response to targeted therapy in acute promyelocytic leukemia. Blood.

[B23] Gari M, Abuzenadah A, Chaudhary A (2008). Detection of FLT3 oncogene mutations in acute myeloid leukemia using conformation sensitive gel electrophoresis. Int J Mol Sci.

[B24] Gregory TK, Wald D, Chen Y (2009). Molecular prognostic markers for adult acute myeloid leukemia with normal cytogenetics. J Hematol Oncol.

[B25] Griffith J, Black J, Faerman C (2004). The structural basis for autoinhibition of FLT3 by the juxtamembrane domain. Mol Cell.

[B26] Ishfaq M, Malik A, Faiz M (2012). Molecular characterization of FLT3mutations in acute leukemia patients in Pakistan. Asian Pac JCancer Prev.

[B27] Lagunas-Rangel FA, Chávez-Valencia V (2017). FLT3-ITD and its current role in acute myeloid leukaemia. Med Oncol.

[B28] Leung AYH, Man C-H, Kwong Y-L (2013). FLT3 inhibition: a moving and evolving target in acute myeloid. Leukemia.

[B29] Levis M (2013). FLT3 mutations in acute myeloid leukemia: what is the best approach in 2013?. Hematol Am Soc Hematol Educ Program.

[B30] Meshinchi S, Woods WG, Stirewalt DL (2001). Prevalence and prognostic significance of Flt3 internal tandem duplication in pediatric acute myeloid leukemia. Blood.

[B31] Patnaik MM (2018). The importance of FLT3 mutational analysis in acute myeloid leukemia. Leuk Lymphoma.

[B32] Pazhakh V, Zaker F, Alimoghaddam K (2011). Detection of nucleophosmin and FMS-like tyrosine kinase-3 gene mutations in acute myeloid leukemia. Ann Saudi Med.

[B33] Rezaei N, Arandi N, Valibeigi B (2017). FMS-Like Tyrosine Kinase 3 (FLT3) and Nucleophosmin 1 (NPM1) in Iranian Adult Acute Myeloid Leukemia Patients with Normal Karyotypes: Mutation Status and Clinical and Laboratory Characteristics. Turk J Haematol.

[B34] Schnittger S, Schoch C, Dugas M (2002). Analysis of FLT3 length mutations in 1003 patients with acute myeloid leukemia: correlation to cytogenetics, FAB subtype, and prognosis in the AMLCG study and usefulness as a marker for the detection of minimal residual disease. Blood.

[B35] Sheikhha MH, Awan A, Tobal K (2003). Prognostic significance of FLT3 1TD and D835 mutations in AML patients. Hematol J.

[B36] Small D (2006). FLT3 mutations: biology and treatment. Hematol Am Soc Hematol Educ Program.

[B37] Stirewalt DL, Kopecky KJ, Meshinchi S (2006). Size of FLT3 internal tandem duplication has prognostic significance in patients with acute myeloid leukemia. Blood.

[B38] Stirewalt DL, Radich JP (2003). The role of FLT3 in haematopoietic malignancies. Nat Rev Cancer.

[B39] Suzuki R, Onizuka M, Kojima M (2007). Prognostic significance of FLT3 internal tandem duplication and NPM1mutations in acute myeloid leukemia in an unselected patient population. Int J Hematol.

[B40] Thiede C, Steudel C, Mohr B (2002). Analysis of FLT3-activating mutations in 979 patients with acute myelogenous leukemia: association with FAB subtypes and identification of subgroups with poor prognosis. Blood.

[B41] Wang L, Lin D, Zhang X (2005). Analysis of FLT3 internal tandem duplication and D835 mutations in Chinese acute leukemia patients. Leuk Res.

[B42] Wang Y, Li Z, He C (2010). MicroRNAs expression signatures are associated with lineage and survival in acute leukemias. Blood Cells Mol Dis.

[B43] Xu F, Taki T, Eguchi M (2000). Tandem duplication of the FLT3 gene is infrequent in infant acute leukemia. Japan Infant Leukemia Study Group. Leukemia.

[B44] Xu YY, Gao L, Ding Y (2012). Detection and clinical significance of FLT3-ITD gene mutation in patients with acute myeloid leukemia. Zhongguo Shi Yan Xue Ye Xue Za Zhi.

[B45] Yamamoto Y, Kiyoi H, Nakano Y (2001). Activating mutation of D835 within the activation loop of FLT3 in human hematologic malignancies. Blood.

[B46] Yanada M, Matsuo K, Suzuki T (2005). Prognostic significance of FLT3 internal tandem duplication and tyrosine kinase domain mutations for acute myeloid leukemia: a meta-analysis. Leukemia.

[B47] Zheng R, Small D (2005). Mutant FLT3 signaling contributes to a block in myeloid differentiation. Leuk Lymphoma.

[B48] Zwaan CM, Meshinchi S, Radich JP (2003). FLT3 internal tandem duplication in 234 children with acute myeloid leukemia: prognostic significance and relation to cellular drug resistance. Blood.

